# Supplementary material for: Myxococcus xanthus Gliding Motors Are Elastically Coupled to the Substrate as Predicted by the Focal Adhesion Model of Gliding Motility
Source: PLoS Comput Biol. 2014 May 8;10(5):e1003619. doi: 10.1371/journal.pcbi.1003619 (PMC4014417; doi:10.1371/journal.pcbi.1003619)
Supplement: Figure S1 — A biophysical representation of the M. xanthus cell as a mass-spring system. (A) Each flexible cell is represented as a connected string of nodes. Circular nodes are similar to the focal adhesion complexes (FAM) or cell wall distortions (HRM) at which cell propulsion force is generated. Circular nodes are spaced apart by rectangular bodies of fixed dimensions. (B) Linear springs join neighboring nodes (circular and rectangular bodies) and maintain the connectivity between the bodies by opposing change in distance between the connected bodies and apply forces () to that effect. (C) An angular spring between three consecutive circular nodes resists bending of the nodes from straight line formation by introducing elastic bending forces (). (D) Cell propulsive forces are applied at circular nodes along the segment joining the next neighbor node in cell travel direction. (E) Adhesive attachments between the node and the substrate are represented by linear springs that introduce a restoration force () on that node when it is displaced from cell's linear axis. (F) Collision forces () act on the nodes that are in direct contact to prevent overlap of bodies. (PDF) [file pcbi.1003619.s001.pdf]

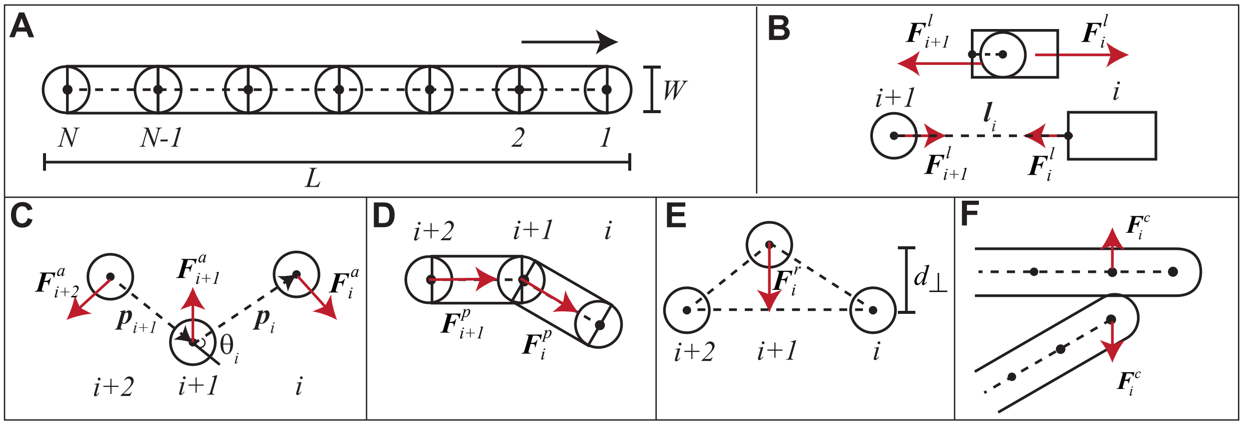

Figure S1: **A biophysical representation of the *M. xanthus* cell as a mass-spring system.** (A) Each flexible cell is represented as a connected string of nodes. Circular nodes are similar to the focal adhesion complexes (FAM) or cell wall distortions (HRM) at which cell propulsion force is generated. Circular nodes are spaced apart by rectangular bodies of fixed dimensions. (B) Linear springs join neighboring nodes (circular and rectangular bodies) and maintain the connectivity between the bodies by opposing change in distance between the connected bodies and apply forces ($\boldsymbol{F}^l$) to that effect. (C) An angular spring between three consecutive circular nodes resists bending of the nodes from straight line formation by introducing elastic bending forces ($\boldsymbol{F}^a$). (D) Cell propulsive forces are applied at circular nodes along the segment joining the next neighbor node in cell travel direction. (E) Adhesive attachments between the node and the substrate are represented by linear springs that introduce a restoration force ($\boldsymbol{F}^r$) on that node when it is displaced from cell's linear axis. (F) Collision forces ($\boldsymbol{F}^c$) act on the nodes that are in direct contact to prevent overlap of bodies.
