# Supplementary material for: Myxococcus xanthus Gliding Motors Are Elastically Coupled to the Substrate as Predicted by the Focal Adhesion Model of Gliding Motility
Source: PLoS Comput Biol. 2014 May 8;10(5):e1003619. doi: 10.1371/journal.pcbi.1003619 (PMC4014417; doi:10.1371/journal.pcbi.1003619)
Supplement: Figure S2 — Quantification of cell-cell collision behavioral data from simulations and experiments. (A) A cell's travel direction is indicated by the vector (– red arrow) pointing from lagging to the leading end. This direction is converted into the cell orientation () by measuring the angle between the cell direction vector () and the horizontal axis () in the anti-clockwise direction. The position of cell collision is identified by the node number of the primary cell where the secondary cell first makes contact. (B) Schematics showing the change in cells' orientations at different instances of time during the collision process. (C) Simulation results show the corresponding change in cell orientations with time. (D) Cell tracking using ImageJ software and MTrackJ plugin. Individual cells (red, green) participating in collision are identified and their leading (H) and lagging (T) ends are marked in consecutive time-lapse images during collision process. The chain of points for each color represents the tracking history of a marked cell's end through the time-lapse images. (E) Orientation of primary (red) and secondary cell (green) as a function of time during a collision event as measured from time-lapse images. Observe that after the collision secondary cell (green line) changes its orientation and aligns with the primary cell (red line). (PDF) [file pcbi.1003619.s002.pdf]

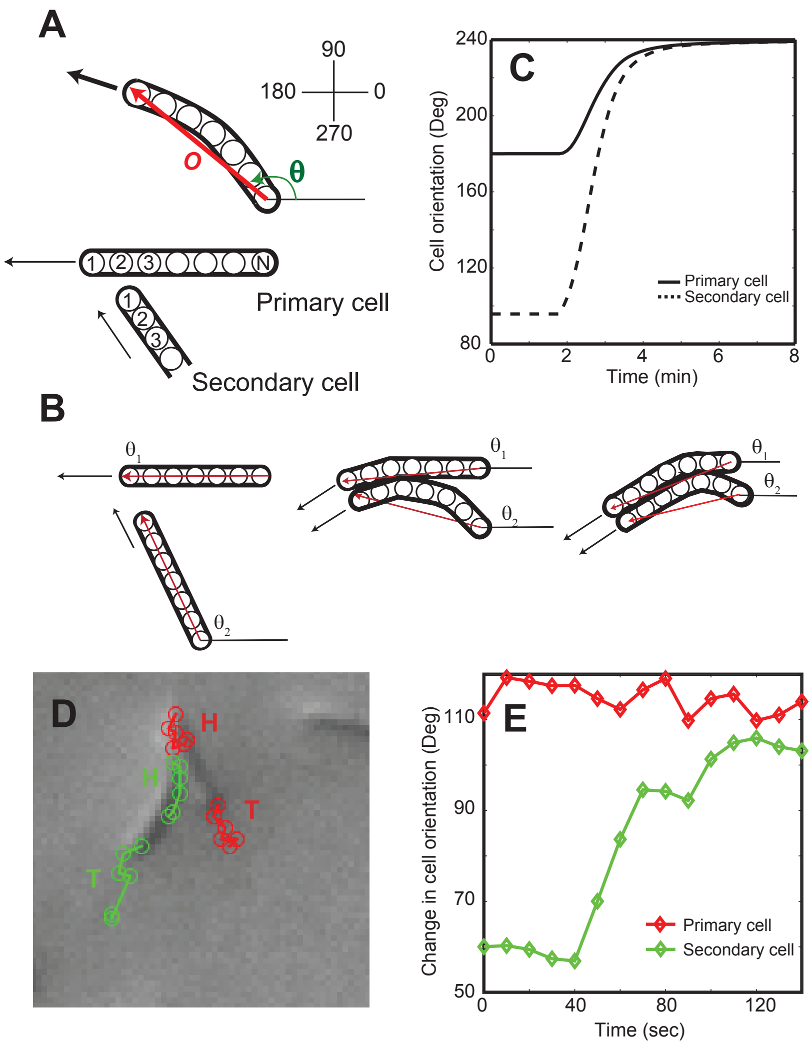

Figure S2: **Quantification of cell-cell collision behavioral data from simulations and experiments.** (A) A cell's travel direction is indicated by the vector (***O*** – red arrow) pointing from lagging to the leading end. This direction is converted into the cell orientation (θ) by measuring the angle between the cell direction vector (***O***) and the horizontal axis (y=0) in the anti-clockwise direction. The position of cell collision is identified by the node number of the primary cell where the secondary cell first makes contact. (B) Schematics showing the change in cells' orientations at different instances of time during the collision process. (C) Simulation results show the corresponding change in cell orientations with time. (D) Cell tracking using ImageJ software and MTrackJ plugin. Individual cells (red, green) participating in collision are identified and their leading (H) and lagging (T) ends are marked in consecutive time-lapse images during collision process. The chain of points for each color represents the tracking history of amarked cell's end through the time-lapse images. (E) Orientation of primary (red) and secondary cell (green) as a function of time during a collision event as measured from time-lapse images. Observe that after the collision secondary cell (green line) changes its orientation and aligns with the primary cell (red line).
