# Supplementary material for: Myxococcus xanthus Gliding Motors Are Elastically Coupled to the Substrate as Predicted by the Focal Adhesion Model of Gliding Motility
Source: PLoS Comput Biol. 2014 May 8;10(5):e1003619. doi: 10.1371/journal.pcbi.1003619 (PMC4014417; doi:10.1371/journal.pcbi.1003619)
Supplement: Figure S3 — Cell collision behavior varies for different collision positions. (A) Change in the primary cell orientation with time for different collision node positions (different colors) from the leading end of the primary cell. (Note that collisions occur around 0.5 min). (B) Absolute change in the primary cell orientation before and after the collision as a function of node position. Data points shown in different colors correspond to the lines in panel A. (PDF) [file pcbi.1003619.s003.pdf]

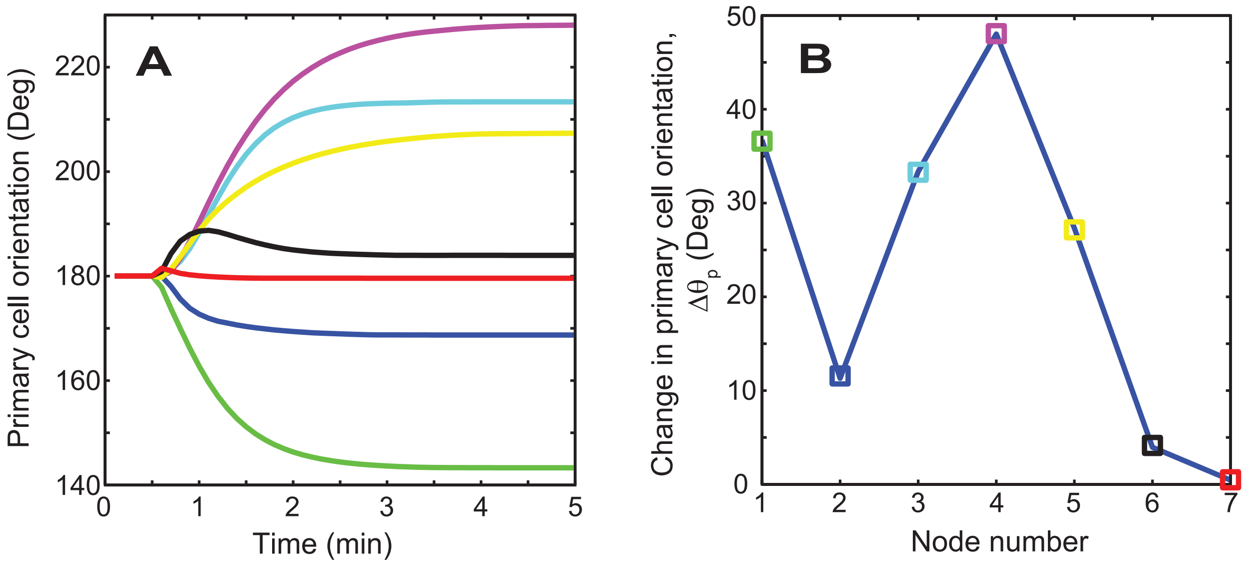

Figure S3: **Cell collision behavior varies for different collision positions** (A) Change in the primary cell orientation with time for different collision node positions (different colors) from the leading end of the primary cell. (Note that collisions occur around 0.5 min). (B) Absolute change in the primary cell orientation before and after the collision as a function of node position. Data points shown in different colors correspond to the lines in panel A.
