# Supplementary material for: Myxococcus xanthus Gliding Motors Are Elastically Coupled to the Substrate as Predicted by the Focal Adhesion Model of Gliding Motility
Source: PLoS Comput Biol. 2014 May 8;10(5):e1003619. doi: 10.1371/journal.pcbi.1003619 (PMC4014417; doi:10.1371/journal.pcbi.1003619)
Supplement: Figure S4 — Cell properties measured in experimental data for wild-type cells. (A) Distribution of collision times from the experimental data of wild-type cells. (B) Tracking history of individual cell orientations over time indicating the spontaneous random cell orientation changes. Each trajectory/color represents measurements from a single isolated cell. (C) The distribution of collision angles and (D) cell lengths from wild-type cell data. (PDF) [file pcbi.1003619.s004.pdf]

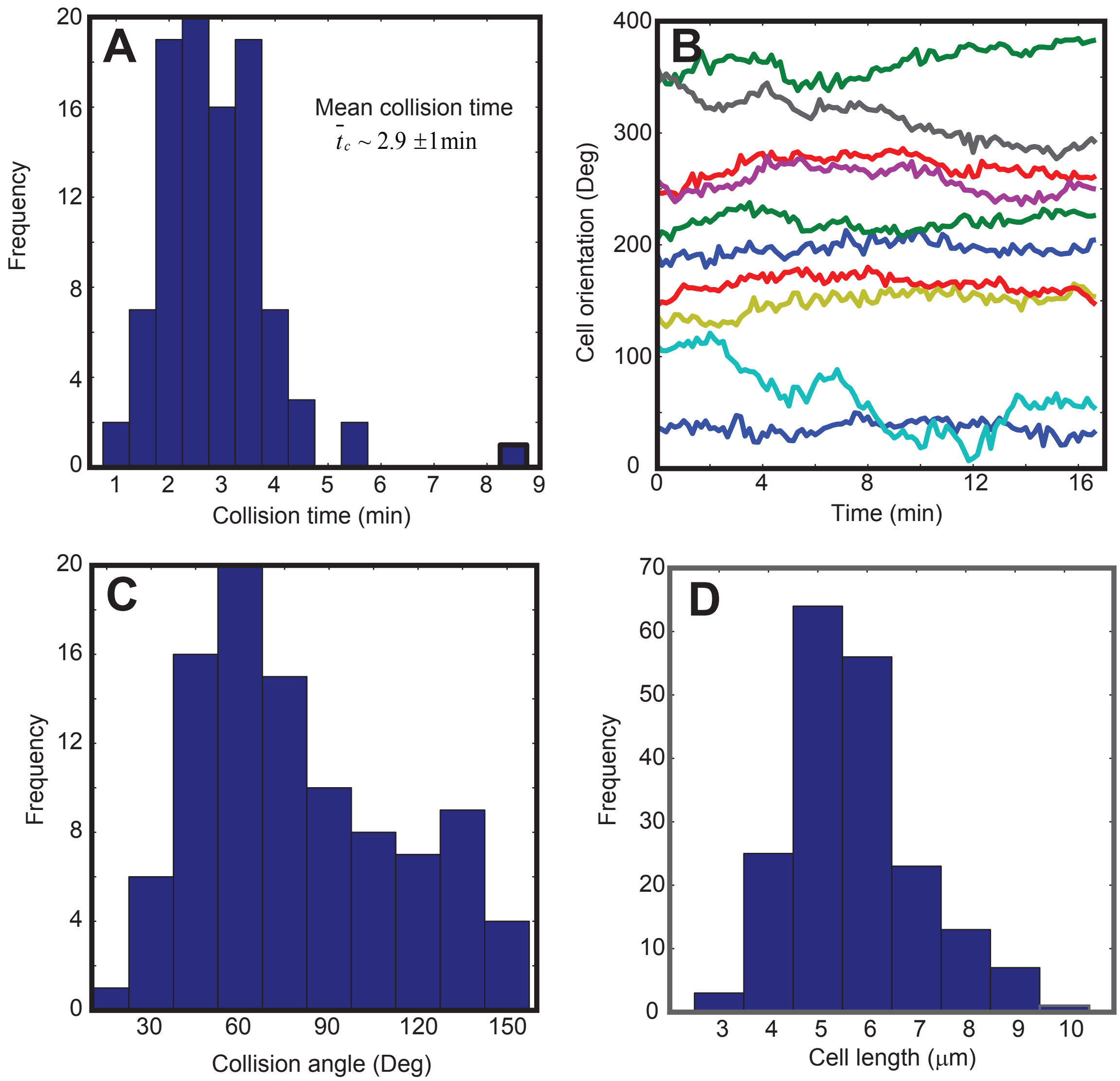

Figure S4: **Cell properties measured in experimental data for wild-type cells.** (A) Distribution of collision times from the experimental data of wild-type cells. (B) Tracking history of individual cell orientations over time indicating the spontaneous random cell orientation changes. Each trajectory/color represents measurements from a single isolated cell. (C) The distribution of collision angles and (D) cell lengths from wild-type cell data.
