# Supplementary material for: Myxococcus xanthus Gliding Motors Are Elastically Coupled to the Substrate as Predicted by the Focal Adhesion Model of Gliding Motility
Source: PLoS Comput Biol. 2014 May 8;10(5):e1003619. doi: 10.1371/journal.pcbi.1003619 (PMC4014417; doi:10.1371/journal.pcbi.1003619)
Supplement: Figure S5 — Cell collision behavior for variations in cell geometrical parameters. (A) Mean and standard deviations in the primary cell orientation changes as a function of cell collision position for variations in the cell mechanical parameters (see Table S2). Black circles represent mean values from the experimental observations. (B) Variations in the maximum change in the primary cell orientation for small perturbation (, ∼half-cell width) of collision position from center of the node. (C) Maximum change in the primary cell orientation in cell collisions as a function of the variation in cell length. Here length of the primary cell is varied while the secondary cell length is fixed at . We also find that the results are similar for variation of secondary cell length (data not shown). (D) Mean and standard deviation in primary cell orientation change as a function of adhesive strength for an increased number of adhesive complexes per cell (9 complexes). (PDF) [file pcbi.1003619.s005.pdf]

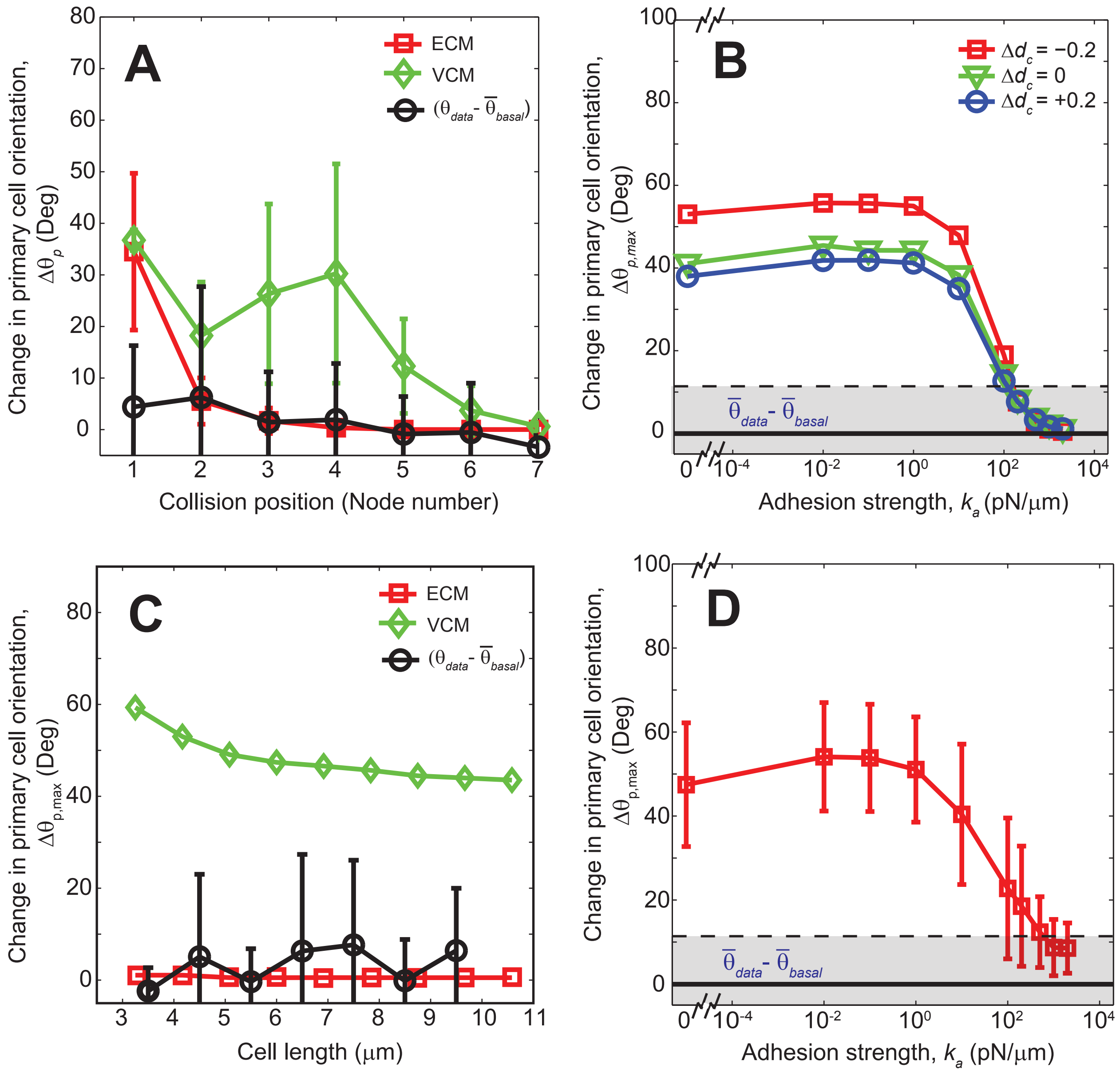

Figure S5: **Cell collision behavior for variations in cell geometrical parameters.** (A) Mean and standard deviations in the primary cell orientation changes as a function of cell collision position for variations in the cell mechanical parameters (see Table S2). Black circles represent mean values from the experimental observations. (B) Variations in the maximum change in the primary cell orientation for small perturbation ($\Delta d_c = \pm 0.2$ μm, ~ half-cell width) of collision position from center of the node. (C) Maximum change in the primary cell orientation in cell collisions as a function of the variation in cell length. Here length of the primary cell is varied while the secondary cell length is fixed at 7 μm . We also find that the results are similar for variation of secondary cell length (data not shown). (D) Mean and standard deviation in primary cell orientation change as a function of adhesive strength for an increased number of adhesive complexes per cell (9 complexes).
