# Supplementary material for: Myxococcus xanthus Gliding Motors Are Elastically Coupled to the Substrate as Predicted by the Focal Adhesion Model of Gliding Motility
Source: PLoS Comput Biol. 2014 May 8;10(5):e1003619. doi: 10.1371/journal.pcbi.1003619 (PMC4014417; doi:10.1371/journal.pcbi.1003619)
Supplement: Figure S6 — Force-velocity relation of M. xanthus gliding motors at various nigericin concentrations. (A–C) Force velocity curves for three different nigericin concentrations: 0 µM (A, blue circles), 10 µM (B, black diamonds), 20 µM (C, red squares). Velocity decreases exponentially with force but never becomes negative consistent with an elastic coupling and inconsistent with a viscous coupling between the bead and motor. The dashed lines are exponential fits to the data. Error bars represent the standard error of the mean across trials (>6 trials per data point). (PDF) [file pcbi.1003619.s006.pdf]

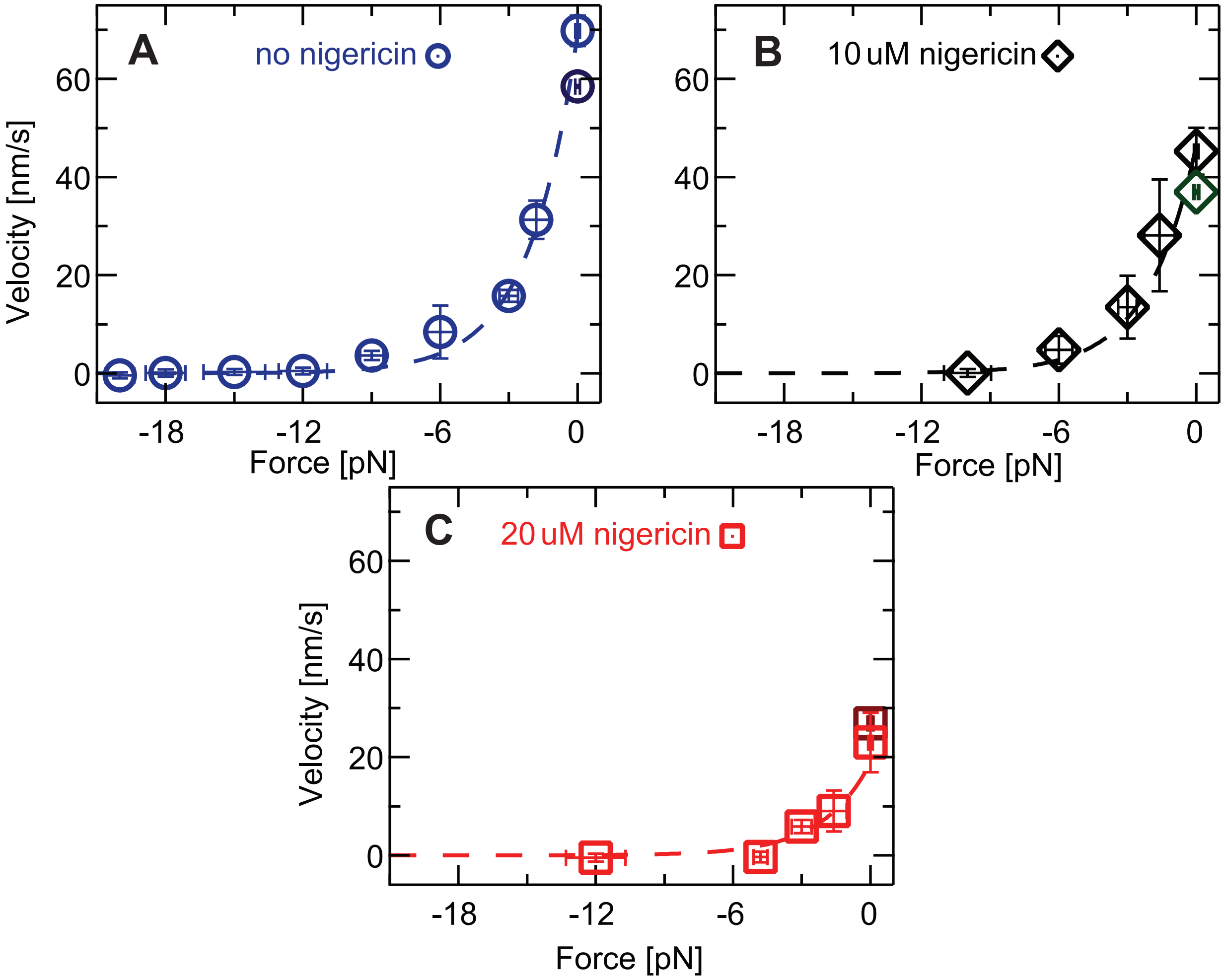

Figure S6: **Force-velocity relation of *M. xanthus* gliding motors at various nigericin concentrations.** (A-C) Force velocity curves for three different nigericin concentrations: 0 µM (A, blue circles), 10 µM (B, black diamonds), 20 µM (C, red squares). Velocity decreases exponentially with force but never becomes negative consistent with an elastic coupling and inconsistent with a viscous coupling between the bead and motor. The dashed lines are exponential fits to the data. Error bars represent the standard error of the mean across trials (> 6 trials per data point).
