# Supplementary material for: Myxococcus xanthus Gliding Motors Are Elastically Coupled to the Substrate as Predicted by the Focal Adhesion Model of Gliding Motility
Source: PLoS Comput Biol. 2014 May 8;10(5):e1003619. doi: 10.1371/journal.pcbi.1003619 (PMC4014417; doi:10.1371/journal.pcbi.1003619)
Supplement: Table S1 — Parameters used in flexible cell model. (PDF) [file pcbi.1003619.s007.pdf]

Table S1: Parameters used in flexible cell model

Symbol	Description	Value
L	Cell length	6.5 µm [4,33]
W	Cell width	0.5 µm [4,33]
N	Number of nodes per cell	7
N_a	Number of adhesion complexes	5 [14,22]
ρ	Cell density	1000 kg/m^3
k_l	Linear spring constant	Managed by Box2D
k_b	Angular spring constant/bending stiffness	10^{-17} N.m [33-35]
k_a	Substrate attachment spring constant	50-2000 pN/µm [20,21]
$L_{\max}$	Bond breaking length	0.5 µm (cell width)
v^f	Mean speed of the cell	4 µm/min [40]
t_{step}	Simulation time step	5×10^{-3} sec
µ	Viscosity of the slime	10^{-3} kg/m.s
F^p	Propulsive force per cell	60 pN
