# Supplementary material for: Myxococcus xanthus Gliding Motors Are Elastically Coupled to the Substrate as Predicted by the Focal Adhesion Model of Gliding Motility
Source: PLoS Comput Biol. 2014 May 8;10(5):e1003619. doi: 10.1371/journal.pcbi.1003619 (PMC4014417; doi:10.1371/journal.pcbi.1003619)
Supplement: Table S2 — Mechanical parameters varied in the model for testing the robustness of model results. (PDF) [file pcbi.1003619.s008.pdf]

Table S2: Mechanical parameters varied in the model for testing the robustness of model results

**Parameter**	**Range varied**
Angular spring constant (k_b)	$10^{-18} – 10^{-16}$ N.m
Spring constant of substrate attachment (k_a)	0 – 2000 pN/µm
Drag coefficient between cell surface and substrate environment	$9\times10^{-5} – 9\times10^{-3}$ kg/s
Bond breaking length ($L_{\max}$)	0.25 – 1.0 µm
